# Screening of GPCR drugs for repurposing in breast cancer

**DOI:** 10.3389/fphar.2022.1049640

**Published:** 2022-12-06

**Authors:** Noor Mazin Abdulkareem, Raksha Bhat, Reid T. Powell, Soumya Chikermane, Soham Yande, Lisa Trinh, Hala Y. Abdelnasser, Mantasha Tabassum, Alexis Ruiz, Mary Sobieski, Nghi D. Nguyen, Jun Hyoung Park, Camille A. Johnson, Benny A. Kaipparettu, Richard A. Bond, Michael Johnson, Clifford Stephan, Meghana V. Trivedi

**Affiliations:** ^1^ Department of Pharmacological and Pharmaceutical Sciences, University of Houston College of Pharmacy, Houston, TX, United states; ^2^ Department of Pharmacy Practice and Translational Research, University of Houston College of Pharmacy, Houston, TX, United states; ^3^ Institute of Bioscience and Technology, Texas A&M University, Houston, TX, United states; ^4^ Department of Pharmaceutical Health Outcomes and Policy, University of Houston, Houston, TX, United states; ^5^ Department of Molecular and Human Genetics, Baylor College of Medicine, Houston, TX, United states

**Keywords:** G protein-coupled receptors, drug discovery, breast cancer, beta blockers, nebivolol, drug repurposing

## Abstract

Drug repurposing can overcome both substantial costs and the lengthy process of new drug discovery and development in cancer treatment. Some Food and Drug Administration (FDA)-approved drugs have been found to have the potential to be repurposed as anti-cancer drugs. However, the progress is slow due to only a handful of strategies employed to identify drugs with repurposing potential. In this study, we evaluated GPCR-targeting drugs by high throughput screening (HTS) for their repurposing potential in triple-negative breast cancer (TNBC) and drug-resistant human epidermal growth factor receptor-2-positive (HER2+) breast cancer (BC), due to the dire need to discover novel targets and drugs in these subtypes. We assessed the efficacy and potency of drugs/compounds targeting different GPCRs for the growth rate inhibition in the following models: two TNBC cell lines (MDA-MB-231 and MDA-MB-468) and two HER2+ BC cell lines (BT474 and SKBR3), sensitive or resistant to lapatinib + trastuzumab, an effective combination of HER2-targeting therapies. We identified six drugs/compounds as potential hits, of which 4 were FDA-approved drugs. We focused on β-adrenergic receptor-targeting nebivolol as a candidate, primarily because of the potential role of these receptors in BC and its excellent long-term safety profile. The effects of nebivolol were validated in an independent assay in all the cell line models. The effects of nebivolol were independent of its activation of β3 receptors and nitric oxide production. Nebivolol reduced invasion and migration potentials which also suggests its inhibitory role in metastasis. Analysis of the Surveillance, Epidemiology and End Results (SEER)-Medicare dataset found numerically but not statistically significant reduced risk of all-cause mortality in the nebivolol group. In-depth future analyses, including detailed *in vivo* studies and real-world data analysis with more patients, are needed to further investigate the potential of nebivolol as a repurposed therapy for BC.

## Introduction

Drug repurposing is an attractive strategy to circumvent the time-consuming and costly drug discovery-development process (Lotfi Shahreza et al., 2018; Xue et al., 2018). Repurposed drugs can be available for clinical use faster because they require only preclinical and clinical efficacy studies without requiring extensive safety studies. Several studies have shown that several Food and Drug Administration (FDA)-approved drugs may be repurposed as anti-cancer drugs in prevention and treatment settings due to their mechanisms of action (Sleire et al., 2017; Hernandez-Lemus and Martinez-Garcia, 2020; Sertedaki and Kotsinas, 2020; Zhang et al., 2020). For example, metformin used to treat diabetes, is found to have anti-cancer effects and may also prevent tumor development, possibly by inhibiting the AMPK/mTOR pathway and immunomodulation (Bodmer et al., 2010; Noto et al., 2012; Franciosi et al., 2013; Wu et al., 2015; Chae et al., 2016; Gong et al., 2016; Higurashi et al., 2016). Moreover, lipid-lowering statins may exhibit anti-cancer properties by targeting the mevalonate pathway *via* disruption of the cell cycle and cell proliferation (Wong et al., 2002; Chan et al., 2003; Boudreau et al., 2010; Stryjkowska-Gora et al., 2015). In addition, non-steroidal anti-inflammatory (NSAID) drugs such as aspirin and COX-2 inhibitors, have anti-cancer activity (Gasic et al., 1972; Elder et al., 1996; Fu et al., 2004; Brown and DuBois, 2005; Cuzick et al., 2009; Mahboubi Rabbani and Zarghi, 2019). Systematic comparison of randomized trials with cohort and case-control studies have shown that the regular use of aspirin is associated with a significant reduction in the incidence of several types of cancers like esophageal, colorectal, biliary, gastric, and breast cancer (BC) (Algra and Rothwell, 2012). However, the progress in identifying drugs with a higher likelihood of repurposing for cancer has been limited due to using only a few strategies such as clinical correlative studies, experimental investigation based on mechanism of action, and computational studies to identify candidate drugs (Nowak-Sliwinska et al., 2019; Zhang et al., 2020).

Given a greater need for better safety of the potential repurposed drugs for cancer therapy, we have taken a unique approach of focusing on drugs binding to G protein-coupled receptors (GPCRs) as targets. With more than 800 receptors in the human genome, GPCRs constitute the largest superfamily of cell surface druggable targets (Sriram and Insel, 2018). About 350 non-olfactory GPCRs are suggested to be druggable targets, and 165 of them are proven drug targets (Yang D. et al., 2021). The recent statistics indicate that 527 FDA-approved drugs and approximately 60 drug candidates presently in clinical trials target different GPCR pathways (Yang D. et al., 2021). GPCR-targeting drugs are often used to treat chronic diseases because of their excellent safety profile (Hauser et al., 2018). GPCRs are overexpressed and involved in several cellular processes in cancer, such as tumor growth, angiogenesis, and metastasis (Dorsam and Gutkind, 2007; Lappano and Maggiolini, 2011; De Francesco et al., 2017). However, most GPCR targets remain unidentified, and only a few FDA-approved drugs targeting GPCRs have been investigated for their anti-cancer effects (Lappano and Maggiolini, 2017).

In this study, we aimed to identify GPCR-targeting drugs for repurposing in triple-negative BC (TNBC) and drug-resistant human epidermal growth factor receptor-2-positive (HER2+) BC due to the unmet clinical need to discover novel targets and drugs (Kolbasnikov, 1987; Dai et al., 2015; Madrid-Paredes et al., 2015; de Melo Gagliato et al., 2016; Goutsouliak et al., 2020). We performed a high throughput screening (HTS) aimed at assessing the efficacy and potency of a large panel of drugs/compounds targeting various GPCRs for the growth rate inhibition in the following models: two TNBC cell lines (MDA-MB-231 and MDA-MB-468) and two HER2+ BC cell lines (BT474 and SKBR3), sensitive or resistant to lapatinib + trastuzumab, an effective combination of anti-HER2 therapies. Here, we report six drugs/compounds as potential hits, 4 of which are already FDA-approved drugs. We selected the β-adrenergic receptor-targeting drug, nebivolol as a candidate primarily due to the potential role of these receptors in BC and the favorable long-term safety profile of β-blockers. Additionally, the effects of nebivolol were validated in an independent assay in all the cell line models. Since metastasis is the main cause of early cancer-related mortality for these BC subtypes, we also investigated the effects of nebivolol on invasion and migration of BC. We found that nebivolol reduced invasion and migration of TNBC cells in a concentration-dependent manner. We also found that the effects of nebivolol were not derived through β3 agonism or nitric oxide (NO) production. We also conducted multivariable Cox proportional hazards modeling using the real-world data from the Surveillance, Epidemiology and End Results (SEER)-Medicare database. There was a reduced but not statistically significant risk of all-cause mortality in the nebivolol groups [adjusted hazard ratio (aHR) of 0.71] compared to the carvedilol group.

## Materials and methods

### Cell lines and reagents

All experiments were conducted using six cell line models: two TNBC cell lines (MDA-MB-231 and MDA-MB-468) and two HER2+ BC cell lines (BT474 and SKBR3), either sensitive (Parental, P) or resistant to lapatinib + trastuzumab (LTR). MDA-MB-231 and MDA-MB-468 cell lines were purchased from Baylor College of Medicine Tissue and Cell Culture Core Laboratory. MDA-MB-231 cells were maintained at 37°C and 5% CO_2_ in Dulbecco’s modified Eagle medium (DMEM) and supplemented with 10% heat-inactivated fetal bovine serum (HI-FBS) and 1% Penicillin-Streptomycin-Glutamine (PSG). MDA-MB-468 cells were maintained in Leibovitz’s L-15 Medium (in free gas exchange with atmospheric air) supplemented with 10% HI-FBS and 1% PSG. The BT474 cell line was obtained from AstraZeneca (Cheshire, United Kingdom) (Arpino et al., 2007), and maintained in DMEM supplemented with 10% HI-FBS and 1% PSG. SKBR3 cells were obtained from Dr. Joe Gray’s lab (Berkeley Lab, Berkeley, CA, United States) and were grown in McCoy’s 5A supplemented with 10% HI-FBS and 1% PSG (Huang et al., 2011; Wang et al., 2011). Cell lines resistant to HER-targeted therapy (Lapatinib + Trastuzumab) (LTR) were obtained from Dr. Rachel Schiff’s lab. These cells were generated by long-term culture of the cells in their original media with increasing concentrations of trastuzumab (1–50 μg/ml) and lapatinib (0.1–1 μM) as described before (Huang et al., 2011; Wang et al., 2011). MCF10A cell line was maintained at 37°C and 5% CO_2_ using MEGM™ Mammary Epithelial Cell Growth Medium BulletKit™ with 100 ng/ml cholera toxin. SUM159 cell line was maintained in DMEM supplemented with 5% HI-FBS and 1% PSG.

### Drugs

Trastuzumab (Herceptin^®^, manufactured by Genentech, San Francisco, CA, United States) was purchased from McKesson and was dissolved in sterile, distilled water provided with the drug. Lapatinib was obtained from LC Laboratories (MA, United States) and was dissolved in sterile dimethyl sulfoxide (DMSO). For the screening, a library of 284 drugs/compounds was purchased from Selleck laboratories. Additional 38 FDA-approved drugs were purchased from Tocris Bioscience, 20 were purchased from MedChemExpress, and 8 were from Sigma (Supplementary File S1). Drug dilutions were made in appropriate media such that the final DMSO concentration was less than 0.1%. Anisomysin and BKM-120 were purchased from Selleck and were used as positive controls. For validation studies, nebivolol, carvedilol and metoprolol were purchased from Selleck laboratories, L-748337 was purchased from Tocris Bioscience, and L-NAME was purchased from MilliporeSigma.

### Growth rate inhibition assay

A concentration-response analysis of 350 GPCR-targeting compounds was conducted using a quantitative HTS (Inglese et al., 2006) in panel of six BC cell lines as described above. The assay conditions (cell number and incubation time) were optimized for each cell line by plating cells at 5 different cell densities and monitoring growth daily for 5 days during assay development. Cell plating densities were selected that ensure cells are in log phase growth over the course of the experiment while remaining at or below 70–80% confluence on the last day of the assay. For primary screens, each assay plate contained a pre-arrayed experimental drug library, 16 positive controls, an 8-point dose response curve in duplicate of BKM120, and 16 DMSO-treated negative controls wells. Additional Day 0 (pre-drug exposure) and Day 3 (untreated endpoint) were also collected from separate plates and were used for the statistical correction of growth. Each assay plate had a single concentration (0.1, 1.0 or 10 μM) of all the drugs on the plate. Assay plates were then fixed with 4% paraformaldehyde (v/v) after a 72-h drug exposure and the nuclei were labeled with Hoechst 33342 or 4,6-Diamidino-2-phenylindole dihydrochloride prior to imaging. All fluorescent cell images were collected using a 4x/0.2NA Plan Apo lens using a GE IN Cell 6,000 Analyzer at 405 nm/455 nm excitation/emission wavelength.

### Selection of candidates

Each candidate drug/compound was marked for every GPCR it had activity against and its mode of action (e.g., agonist, partial agonist, inverse agonist, or antagonist) using The International Union of Basic and Clinical Pharmacology (IUPHAR)/British Pharmacological Society guide to pharmacology website (Database version 2021.3 August 2022, https://www.guidetopharmacology.org/) (Supplementary File S1). The compounds not found to have direct activity against GPCRs, were excluded from the analysis. We used two cut-offs to identify hits: 1) area under the curve for growth rate inhibition (AUC_GRI_) of <0.95 and 2) concentration by which 50% of growth inhibition is achieved (GR_50_) of <10 μM in two TNBC and two LTR cell lines. FDA-approved GPCR-targeting drugs used for chronic diseases were identified.

### Validation studies using 8-point concentration-response curve

To validate the primary HTS results in panel of six BC cell lines as described above, 8-point concentration-response curves were generated. Briefly, cells were seeded at 4,000–6,000 cells/well in a 96-well tissue culture plate for overnight attachment. Then, drugs were added at various concentrations (10 nM–31.6 μM) for 72 h. The plates were scanned using the EnSight Multimode Plate Reader equipped with well-imaging technology (PerkinElmer, MA, United States). Cell count was obtained by digital phase and brightfield imaging. Data was normalized to vehicle 1) and plotted and analyzed using GraphPad Prism version 9. For determination of IC_50_ values, the data was fitted using non-linear regression analysis and 3-parameter logistic equation with the slope set to 1: Y = Bottom + ((Top–Bottom)/(1 + 10^ ((X–LogIC_50_))).

### Invasion and migration assays

Invasion and migration assays were performed with or without Matrigel, respectively, on inserts as described before (Bhat et al., 2018).

### Seahorse assay

To confirm the OXPHOS inhibitory function of Nebivolol, SUM159 TNBC cells (15,000 cells/well) were seeded onto XFp Seahorse cell plate and treated with 10 μM nebivolol. The oxygen conception rate (OCR) was measured using Cell Mito Stress kit (Cat. 103010-100, Agilent Technologies) in a Seahorse XFp Extracellular Flux Analyzers (Agilent Technologies) according to manufacturer’s instructions and as described before (Park et al., 2016).

### Statistical analyses

All cell-based studies were run at least in duplicates and repeated at least two independent times. All data points of cell numbers at different time points (0 vs. 72 h) and under various conditions (vehicle vs. different concentrations of drugs) were used for analysis. The number of nuclei present in each image was counted using *IN Cell Developer software version* 9.2. Cell growth was determined using the pre-treatment, negative control of the endpoint, and the statistical methods described by the National Cancer Institute (Holbeck et al., 2010; Hafner et al., 2016). Numeric data was analyzed using *Pipeline Pilot version 9.5* and GraphPad Prism version 9 to determine the fitness and the level of statistical significance of the assays. To evaluate plate-to-plate variability, data from on-plate controls were used to compare the minimum significant ratio (MSR) (NCGC Assay Guidance Manual) (Coussens et al., 2018). The quality of assay was assessed using the robust z-prime statistics as previously described (Zhang et al., 1999).

### Analysis of the SEER-Medicare data

A retrospective cohort study was conducted using multi-year SEER-Medicare data from 2009 to 2015 in patients older than 66 years of age. The cohort included patients with BC as the 1st or only cancer between 01/01/2010 and 12/31/2014, continuously enrolled in Medicare parts A, B, and D during the 6 months immediately prior to BC diagnosis, received β-blocker monotherapy (carvedilol, metoprolol, or nebivolol) for at least 6 months prior to BC diagnosis without a gap of more than 30 days in therapy. Patients who used multiple β-blockers, enrolled in Health Maintenance Organization (HMO) or Part C during the 6-month baseline period, or those eligible for Medicare due to reasons other than age, were excluded. Patients were categorized into mutually exclusive and collectively exhaustive cohorts based on the type of β-blocker they used. Covariates like age, sex, race, ethnicity, BC as the first cancer, stage of BC, subtype of BC, use of statins due to their effects on BC mortality (Lv et al., 2020; Hosio et al., 2021; Kim et al., 2022; Zhao et al., 2022), Charlson comorbidity index, and the year of BC diagnosis were measured. The all-cause mortality and BC mortality were the outcomes for this study, and patients were followed from incident BC diagnosis until the earliest of all-cause mortality, discontinuation of the index β-blocker, switching or concomitant use of comparator β-blockers, or the end of the study period (31 December 2015). A sub analysis in patients with TNBC or HER2+ BC subtypes was also conducted. Descriptive statistics were calculated across the exposure groups, mean and standard deviation (sd) was calculated for continuous variables and the frequency and percentages were calculated for categorical variables. The competing risk regression model, adjusted for potential confounding variables, was used to study the association between the β-blocker groups and the risk of all-cause or BC mortality. The Cox proportional hazards model was used to assess the risk of all-cause mortality between the carvedilol, metoprolol, and nebivolol groups, after adjusting for baseline covariates.

## Results

### GPCRs targeting drugs/compounds classification

Out of 350 drugs/compounds, 216 targeted at least one GPCR based on the IUPHAR/BPS guide to pharmacology website (Database version 2021.3, 2 September 2021) (Supplementary File S1). A total of 85 GPCRs were targeted by at least one drug/compound. The most targeted GPCRs (with ≥20 drugs/compounds) were H1, D2, 5-HT2A, 5-HT1A, 5-HT6, α1D, α1A, β2.

### Identification of hits and a candidate compound

We conducted a cell-count based high-throughput growth assay. All screens were completed in 2 biological replicates run per cell line for all of the compounds, and multiple assay quality and reproducibility metrics were monitored throughout the screening campaign. From this analysis, we showed consistent rates of growth in control wells across all runs and cell line models, a high level of reproducibility in dose response curves across biological batches (Running MSR ≤3 for all cell lines), and a highly robust assay read-out determined by the (Median Z′-factor ≥ 0.70 for all cell lines) (Supplementary File S2). Concentration response curves were then evaluated (Supplementary File S3) to calculate AUC_GRI_ and GR_50_ values (Supplementary File S4). Using two cut-offs of <0.95 AUC_GRI_ and <10 μM GR_50_ values in two TNBC and two LTR cell lines, we identified six hits in all BC cell lines models (Table 1). Out of these six candidate drugs/compounds, 4 were FDA approved drugs that targeted CaS, mGlu5, β1, β2, β3, 5-HT2A, 5-HT1A, 5-HT1B, 5-HT5A, 5-HT7, and 5-HT6, receptors, with some drugs targeting more than one GPCRs (Table 1). Among these targets, β-adrenergic receptors were identified as commonly targeted GPCRs for various chronic diseases like heart failure and hypertension (Yang A. et al., 2021; Abosamak and Shahin, 2022)**,** and with a role in BC (Barron et al., 2012). β-blockers are typically well tolerated drugs and are used long-term by many patients. Among β-blockers, only nebivolol (β1, β2, and β3 antagonist) inhibited the growth rate of all cell line models with AUC_GRI_ < 0.95 and GR_50_ < 10 μM (Table 2). Other β-blockers targeting β1 and/or β2 did not inhibit HER2+ BC and TNBC cell growth (Table 2), suggesting that the effects of nebivolol are independent of its β-blocker role. Nebivolol also had more favorable safety profile when compared with other FDA approved candidates (Supplementary File S5), further justifying its selection for further exploration.

**TABLE 1:** AUC_GRI_ and GR_50_ values for candidate drugs/compounds in BC cell lines models.

Drug/compound	TNBC-1		TNBC-2		HER2+BC Resistant-1		HER2+BC Sensitive-1		HER2+BC Resistant-2		HER2+BC Sensitive-2		GPCR target
	MDA-MB-231		MDA-MB-468		BT474-LTR		BT474-P		SKBR3-LTR		SKBR3-P		
	AUC_GRI_	GR_50_ (µM)	AUC_GRI_	GR_50_ (µM)	AUC_GRI_	GR_50_ (µM)	AUC_GRI_	GR_50_ (µM)	AUC_GRI_	GR_50_ (µM)	AUC_GRI_	GR_50_ (µM)	
Cinacalcet HCl^a^	0.93	4.40	0.90	4.14	0.92	3.63	0.90	3.98	0.88	2.08	0.91	3.99	(CaS, mGlu5) ^Ag^
JTC-801	0.71	1.62	0.72	1.57	0.74	2.04	0.75	1.46	0.80	2.34	0.78	2.01	NOP ^Antg^
Nebivolol^a^	0.87	2.84	0.80	2.37	0.88	2.68	0.88	3.30	0.85	4.26	0.94	NA	(β1, β2, β3) ^Antg^
Pimavanserin^a^	0.87	3.26	0.89	3.89	0.94	3.45	0.87	2.67	0.88	2.17	0.86	2.64	5-HT2A^InAg^
SB 225002	0.69	0.46	0.83	1.98	0.73	0.73	0.76	0.94	0.66	0.55	0.80	1.51	CXCR2 ^Antg^
Vortioxetine^a^ (Lu AA21004) HBr	0.88	3.14	0.89	3.38	0.91	3.30	0.87	2.81	0.93	2.48	0.87	3.00	(5-HT1A, 5-HT1B) ^PaAg^, (5-HT5A, 5-HT7, 5-HT2A, 5-HT6) ^Antg^

Ag, Agonist; Antg, Antagonist; InAg, Inverse Agonist; PaAg, Partial Agonist.

^a^ FDA-Approved, NA: not available.

**TABLE 2:** AUC_GRI_ and GR_50_ values for β-blockers in BC cell lines models.

Drug/compound	TNBC-1		TNBC-2		HER2+BC Resistant-1		HER2+BC Sensitive-1		HER2+BC Resistant-2		HER2+BC Senitive-2		GPCR target
	MDA-MB-231		MDA-MB-468		BT474-LTR		BT474-P		SKBR3-LTR		SKBR3-P		
	AUC_GRI_	GR_50_ (µM)	AUC_GRI_	GR_50_ (µM)	AUC_GRI_	GR_50_ (µM)	AUC_GRI_	GR_50_ (µM)	AUC_GRI_	GR_50_ (µM)	AUC_GRI_	GR_50_ (µM)	
Acebutolol HCl	1.03	NA	1.03	NA	1.11	NA	1.05	NA	1.07	NA	1.04	NA	β1 ^Antg^
Timolol Maleate	1.03	NA	1.04	NA	1.11	NA	1.04	NA	1.06	NA	1.02	NA	β2 ^Antg^
Betaxolol	1.05	NA	1.01	NA	1.07	NA	1.01	NA	1.07	NA	1.00	NA	(β1, β2) ^Antg^
Betaxolol hydrochloride (Betoptic)	1.05	NA	1.04	NA	1.10	NA	1.02	NA	1.06	NA	0.98	NA	(β1, β2) ^Antg^
Carteolol HCl	1.04	NA	1.03	NA	1.11	NA	1.05	NA	1.10	NA	1.04	NA	(β1, β2) ^Antg^
Carvedilol	1.03	NA	0.94	3.60	1.04	NA	0.96	7.95	1.07	NA	1.00	NA	(β1, β2) ^Antg^
Metoprolol Tartrate	1.01	NA	0.98	NA	1.01	NA	0.99	NA	1.00	NA	1.00	NA	(β1, β2) ^Antg^
Sotalol	1.03	NA	1.05	NA	1.09	NA	1.05	NA	1.07	NA	1.03	NA	(β1, β2) ^Antg^
ICI-118551	1.06	NA	1.05	NA	1.11	NA	1.04	NA	1.07	NA	1.01	NA	β2 ^InAg^, β3 ^Antg^
Propranolol HCl	1.04	NA	1.03	NA	1.10	NA	1.11	NA	1.06	NA	1.01	NA	(β1, β2) ^Antg^
Labetalol HCl	1.06	NA	1.04	NA	1.07	NA	1.10	NA	1.06	NA	1.01	NA	(β1, β2) ^Antg^
Nebivolol	0.87	2.84	0.80	2.37	0.88	2.68	0.88	3.30	0.85	4.26	0.94	NA	(β1, β2, β3) ^Antg^

Antg, Antagonist; InAg, Inverse Agonist, NA, not available.

### Validation of the candidate compound nebivolol

**FIGURE 1:**
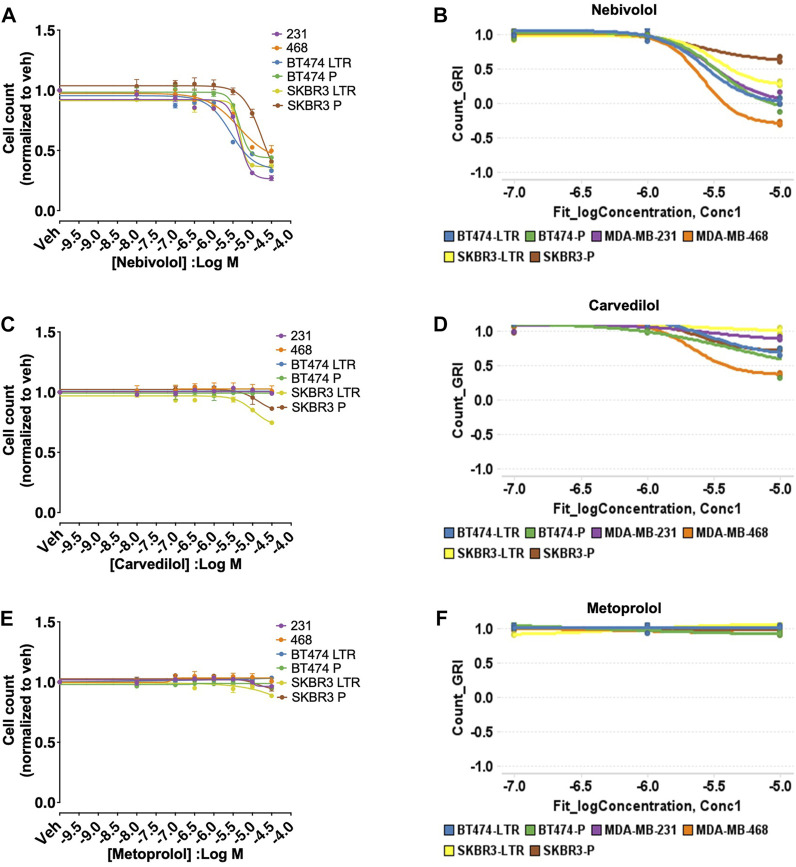
HTS and validation studies indicate that nebivolol inhibits cell growth in all six BC cell lines models. **(A)** Effects of nebivolol on cell growth measured by the cell count in all six BC cell lines models with IC_50_ 6.57 µM (MDA-MB-231), 4.60 µM (MDA-MB-468), 3.47 µM (BT474 LTR), 7.96 µM (BT474 P), 8.05 µM (SKBR3 LTR), 2.55 µM (SKBR3 P). **(B)** HTS data for nebivolol with similar effects on cell growth in all six BC cell lines models. **(C–F)** Carvedilol and metoprolol effects on cell growth measured by the cell count and from HTS data in all six BC cell lines models.

Nebivolol inhibited cell growth measured by the cell count in concentration-dependent manner in all six BC cell lines models (IC_50_ = MDA-MB-231: 6.57 µM, MDA-MB-468: 4.60 µM, BT474 LTR: 3.47 µM, BT474 P: 7.96 µM, SKBR3 LTR: 8.05 µM, SKBR3 P: 2.55 µM, Figure 1A) similar to our screen data (Figure 1B). In addition, carvedilol and metoprolol showed no significant inhibition in agreement with the HTS data (Figures 1C–F). Nebivolol did not inhibit cell growth of the normal human mammary epithelial cell line MCF10A (Supplementary File S6), suggesting cancer cell-specific effects of nebivolol.

**FIGURE 2:**
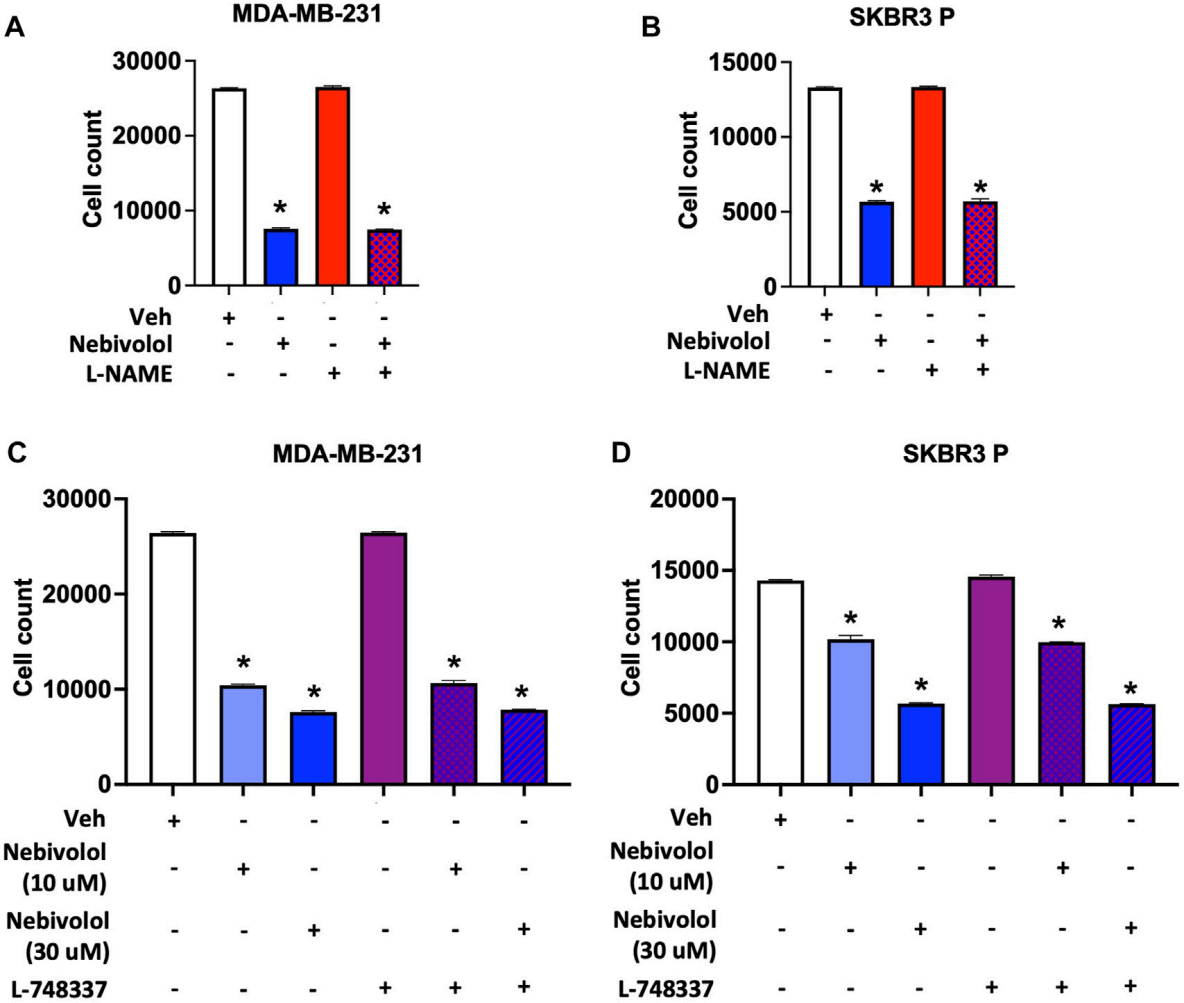
Nitric oxide (NO) synthase inhibitor (L-NAME) and β3 antagonist (L-748337) do not affect nebivolol-induced inhibition of cell growth in BC cells. Effects of L-NAME (1 mM) on nebivolol (10 µM)-induced inhibition of cell growth measured by the cell count in **(A)** MDA-MB-231 and **(B)** SKBR3 P cells. Effects of L-748337 (7 µM) on nebivolol (10 µM or 30 µM)-induced inhibition of cell growth measured by the cell count in **(C)** MDA-MB-231 and **(D)** SKBR3 P cells. * indicates statistically significant difference compared to Veh; *p* < 0.05 by unpaired *t*-test (*n* = 3).

Apart from its β-adrenergic receptors blockade, nebivolol also dilates blood vessels through the L-arginine/NO pathway in the endothelium through β3 agonism (Maffei et al., 2007; Gupta and Wright, 2008; Maffei and Lembo, 2009; Coats and Jain, 2017). Therefore, we tested the effects of β3 antagonist (L-748337, 7 µM) (Rozec et al., 2009) and NO synthase blocker (L-NAME, 1 mM) (Martin et al., 1993) on cell growth by nebivolol in MDA-MB-231 and SKBR3 P cell lines. L-748337 and L-NAME did not reverse the cell growth inhibition effects of nebivolol (Figure 2), suggesting that the effects of nebivolol on cell growth inhibition were independent of β3 receptors and NO pathways.

**FIGURE 3:**
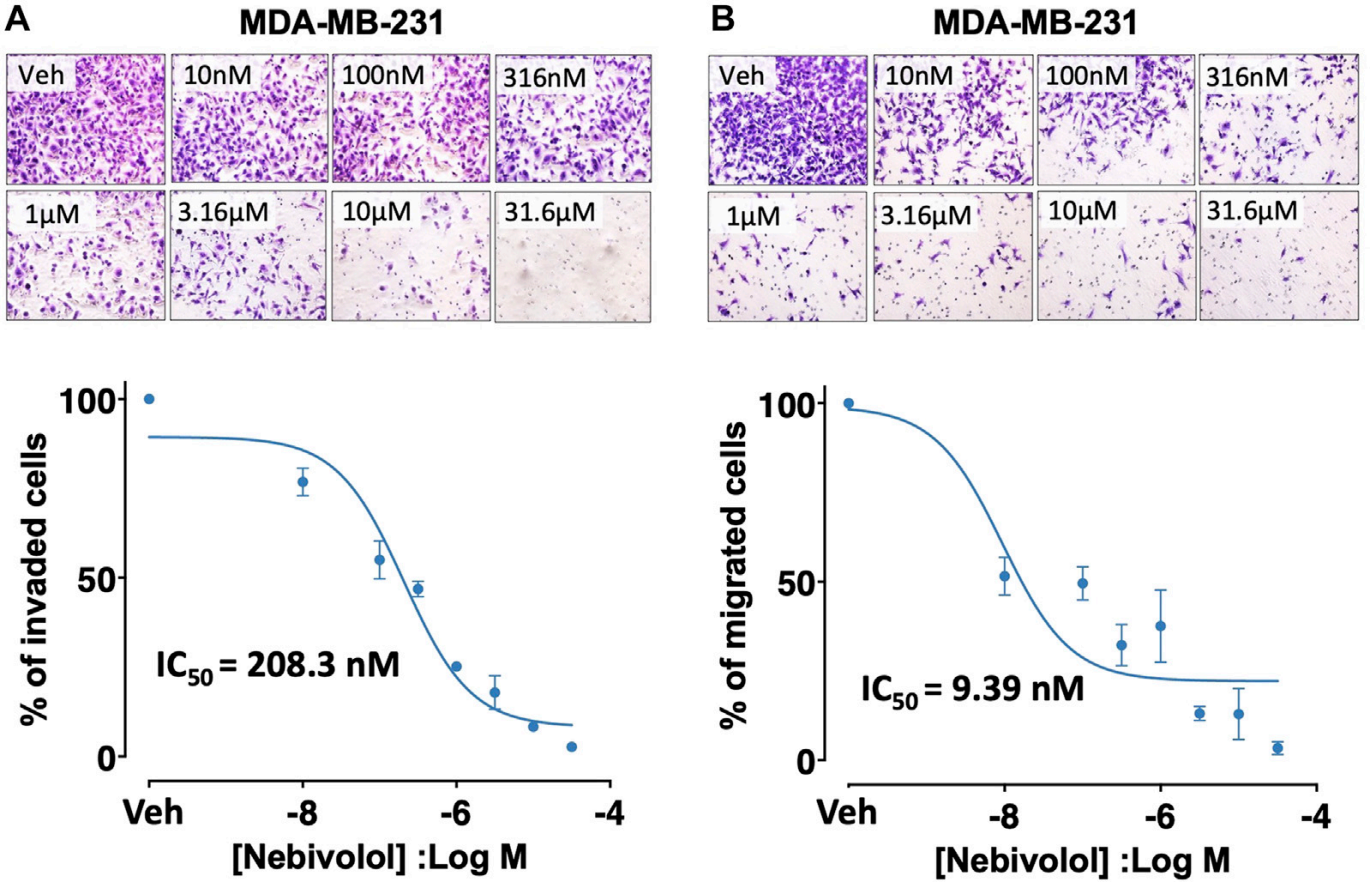
Effects of nebivolol on invasion and migration potentials in MDA-MB-231 cells. Nebivolol (1 nM–31.6 µM) effects on **(A)** invasion and **(B)** migration potentials in MDA-MB-231 cells.

Nebivolol reduced invasion (IC_50_ = 208.3 nM) and migration (IC_50_ = 9.39 nM) of MDA-MB-231 cells in a concentration-dependent manner (Figure 3). Nebivolol (10 µM) also inhibited mitochondrial oxidative phosphorylation (OXPHOS) in TNBC cells (Supplementary File S7), which is consistent with another published report on nebivolol (Nuevo-Tapioles et al., 2020) and high OXPHOS activity of many metastatic TNBC cells (Park et al., 2016; Jia et al., 2018).

### Effects of nebivolol and other β-blockers on all-cause and BC mortality in patients

The cohort included 4,843 patients, of these 4.79% (*n* = 232) patients were in the nebivolol group, 20.42% (*n* = 989) patients were in the carvedilol group, and 74.79% (*n* = 3,622) patients in the metoprolol group. The mean age of patients in the nebivolol group was 76.54 years (sd = 6.67 years), 78.71 years (sd = 7.60 years) in the carvedilol group, and 77.93 years (sd = 7.59 years) in the metoprolol group. Almost 99% of the cohort were females as expected for the BC study. Most of the patients were White and 95% of the cohort were not of Hispanic descent. Most patients were diagnosed at BC stages 0, 1, or 2 across all three β-blockers: 82.76% (*n* = 192) in the nebivolol group, 79.07% (*n* = 782) in carvedilol group, and 81.53% (*n* = 2,953) in metoprolol group. TNBC and HER2+ BC, accounted for 37.93% (*n* = 51) in the nebivolol group, 35.79% (*n* = 180) in the carvedilol group, and 37.71% (*n* = 681) in the metoprolol group. Baseline characteristics of patients are reported in Table 3.

**TABLE 3:** Difference in baseline characteristics between the nebivolol, metoprolol, and carvedilol groups.

Variables	Nebivolol		Metoprolol		Carvedilol		*p*-value
	mean/n	sd/%	mean/n	sd/%	mean/n	sd/%	
Age (mean, sd)	76.54	6.67	77.92	7.6	78.71	7.6	<0.01
Age categories (n, %)							<0.01
65-70	45	19.4	689	19.02	165	16.68	
71-75	71	30.6	870	24.02	216	21.84	
>75	116	50	2063	56.96	608	61.48	
Race (n, %)							<0.01
White	<11	<11	3177	87.71	816	82.51	
Black	<11	<11	252	6.96	110	11.12	
Other	<11	<11	171	4.72	<11	<11	
Missing	<11	<11	22	0.61	<11	<11	
Ethnicity (n, %)							<0.01
Not Hispanic/Latino	<11	<11	3464	95.64	914	92.42	
Hispanic/Latino	<11	<11	158	4.36	75	7.58	
Index Year (n, %)							<0.01
2010	12	5.17	659	18.19	166	16.78	
2011	34	14.66	665	18.36	154	15.57	
2012	57	24.57	682	18.83	211	21.33	
2013	60	25.86	784	21.65	229	23.15	
2014	69	29.74	832	22.97	229	23.15	
Number of Charlson comorbidities (n, %)							<0.01
0	89	38.36	1429	39.45	209	21.13	
1–3	110	47.41	1869	51.6	573	57.94	
4	33	14.22	324	8.95	207	20.93	
Statin use (n, %)	142	61.21	2163	59.72	671	67.85	<0.01
Breast cancer characteristics							
Breast cancer subtype (n, %)							0.44
HER2+	13	5.6	193	5.33	44	4.45	
HR+/HER2-	144	62.07	2256	62.29	635	64.21	
TNBC	16	6.9	269	7.43	69	6.98	
Triple positive	22	9.48	219	6.05	67	6.77	
Unknown	37	15.95	685	18.91	174	17.59	
Breast cancer as the: (n, %)							0.7
First cancer	24	10.34	431	11.9	122	12.34	
Only cancer	208	89.66	3191	88.1	867	87.66	
Breast cancer stage (n, %)							<0.01
Stage 0	35	15.09	493	13.61	119	12.03	
Stage 1	110	47.41	1532	42.3	351	35.49	
Stage 2	47	20.26	928	25.62	312	31.55	
Stage 3	18	7.76	274	7.56	98	9.91	
Stage 4	<11	<11	180	4.97	51	5.16	
Unknown	<11	<11	215	5.94	58	5.86	

For cells with counts <11, in addition to the respective cell, another cell within the category for the exposure group is blinded to prevent calculation of the cell for which count is less than 11.

**FIGURE 4:**
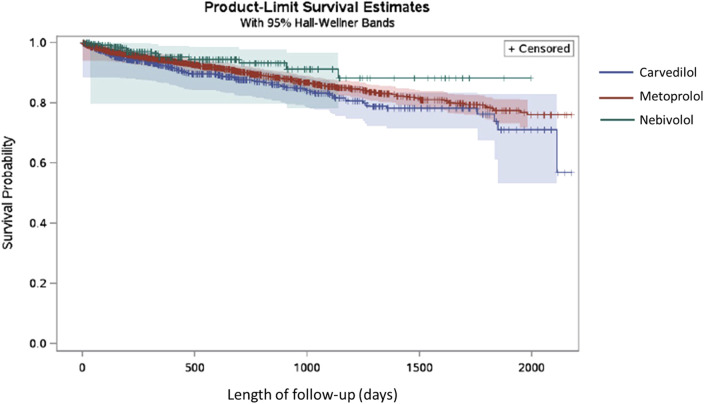
Kaplan Meier plot for nebivolol, carvedilol and metoprolol exposure groups and the time to all-cause mortality in TNBC and HER2+ BC patients.

In the BC cohort, patients using carvedilol (12.74%) had the highest rate of all-cause mortality during the follow-up period, followed by metoprolol (10.77%), and nebivolol (5.60%) of nebivolol. The median time to all-cause mortality in the BC cohort was 306 days for nebivolol, 319.5 days for carvedilol, and 388 days for metoprolol. The median time to BC mortality in this cohort was 250 days for nebivolol, 127.5 days for carvedilol, and 197 days for metoprolol. In the TNBC and HER2+ BC cohort, BC mortality accounted for 7.22% of carvedilol users, and 7.93% of metoprolol users. The event frequency in the nebivolol group was very low and thus the risk of BC mortality in this group could not be assessed using the existing SEER-Medicare data. The median time to BC mortality in the TNBC and HER2+ BC subgroup was 306 days for nebivolol, 386 days for carvedilol, and 281.5 days for metoprolol. The Kaplan Meier plot for the three exposure groups and the time to all-cause mortality is shown in Figure 4. The multivariable Cox proportional hazards model found that there was no significant difference in the risk of all-cause mortality in the nebivolol aHR = 0.71, 95%, confidence interval (CI) = 0.40 to 1.28, *p*-value = 0.25), and metoprolol groups aHR = 1.00, 95%, CI = 0.82 to 1.24, *p*-value = 0.97) compared to the carvedilol group. The multivariable competing risk model found that there was no significant difference in the risk of BC mortality in the metoprolol group (aHR = 1.66, 95% CI = 0.86 to 3.18, *p*-value = 0.13) compared to the carvedilol group (data not shown). The nebivolol group could not be included in this analysis because of low event frequency.

## Discussion

In the present study, we conducted HTS aimed at evaluating the efficacy and potency of GPCR-targeting drugs/compounds for their growth inhibition in TNBC and HER2+ BC cell line models. We identified six drugs/compounds as potential hits. Out of these six hits, 4 were FDA-approved drugs. We selected β-adrenergic receptor-targeting nebivolol as a candidate mainly because of the potential role of these receptors in BC and the favorable long-term safety profile of β-blockers. The effects of nebivolol were validated in an independent assay in all the cell line models. We found that the effects of nebivolol were not derived through β3 agonism or NO production. Nebivolol also reduced invasion/migration potential, suggesting its inhibitory role in metastasis which requires further investigation. Results from the real-world evidence study using the data from the SEER-Medicare data did not find a significant difference in all-cause mortality or BC mortality. However, lab findings from this study highlight the need to identify the exact mechanism of action of nebivolol and the impact of nebivolol therapy on BC mortality in a greater number of patients, especially in TNBC and HER2+ BC subgroups.

The development of new drugs is a lengthy process that is both time and resource consuming. Since it is known that up to 90% of drugs fail during development (Takebe et al., 2018; Hingorani et al., 2019), drug repurposing offers an alternative approach that allows the use of already approved drugs to treat diseases beside previously intended ones (Low et al., 2020). Because the safety of the original drugs have already been extensively assessed and approved, drug repurposing is associated with lower overall developmental costs and risk assessments (Xue et al., 2018; Parvathaneni et al., 2019). We found that the approach to identify GPCR candidates by HTS was highly feasible because HTS assays and large compound libraries with GPCR-targeting chemistry are largely available (Yasi et al., 2020). Previous studies have developed and used HTS platforms to identify new hits in different types of cancers and diseases (Marciano et al., 2019; She et al., 2021; Zhao et al., 2021). To our knowledge, ours is the first study evaluating the effects of only GPCR-targeting drugs/compounds in a panel of cancer cell lines.

Several preclinical studies have suggested that β-blockers play a role in inhibiting various cellular processes involved in BC development and metastasis (Barron et al., 2012). For instance, stress and adrenergic activation was shown to increase proliferation, invasion and migration of BC cells, and these effects were inhibited by β-blockers (Sloan et al., 2010; Wilson et al., 2015; Montoya et al., 2017). Also, β-blockers were found to present a direct cytotoxic activity against BC cancer cells (Szewczyk et al., 2012; Ashrafi et al., 2017). It was found that β-blockers can also increase the production of inflammatory cytokines and inhibit angiogenesis in the tumoral stroma, which may improve the effects of anti-cancer treatments (Pasquier et al., 2011; Jean Wrobel et al., 2016; Caparica et al., 2020). The preclinical evidence of β-blockers activity against BC and the convenient safety profile have generated an interest in repurposing of these drugs in the treatment of BC (Ishida et al., 2016). Few retrospective studies have shown that the use of β-blockers is associated with better prognosis in BC patients (Powe et al., 2010; Spera et al., 2017). However, meta-analyses of studies that integrated patients with both early and advanced stage BC showed that the effects of β-blockers on patient outcomes remain inconclusive (Raimondi et al., 2016; Kim et al., 2017; Li et al., 2020; Caparica et al., 2021). While preclinical studies suggest that β-blockers may be effective in BC, clinical studies investigating associations between the use of β-blockers and better BC outcomes have not tested the effects of individual β-blockers with unique pharmacology.

IUPHAR reports nebivolol as a third generation β-blocker that can inhibit β1, β2, β3 adrenergic receptors (Frazier et al., 2011; Priyadarshni and Curry, 2022). It is primarily classified as β1 adrenergic receptor with the highest affinity among all β-blockers, which explains its good tolerability in patients with lung conditions like asthma (Fongemie and Felix-Getzik, 2015). At doses of ≤10 mg, nebivolol is more selective to β1 compared to β3. Conversely, at higher doses and in patients with poor or impaired metabolism, nebivolol blocks β1 and β2 receptors at similar selectivity (Priyadarshni and Curry, 2022). In addition, nebivolol shows vasodilatory properties *via* endothelium-derived NO induction, mainly by enhancing endothelial NO synthase activity, through β3 agonism (Rozec et al., 2009; Gauthier and Trochu, 2010; Sanaee and Jamali, 2014; Fongemie and Felix-Getzik, 2015). These reports by multiple independent investigators are in contrast to nebivolol labeled as β3 antagonist by IUPHAR. Nonetheless, we found that the effects of nebivolol were not dependent on β3 adrenergic receptors or NO synthase activity. Recently, nebivolol was found to inhibit the growth of colon and breast carcinomas by reducing oxidative phosphorylation *via* blocking Complex I and ATP synthase activities and induction of apoptosis (Nuevo-Tapioles et al., 2020), which was also confirmed in another TNBC cell line by our studies. Hence, nebivolol could act independently of β-adrenergic receptor inhibition.

In conclusion, our HTS and validation data suggest that nebivolol may inhibit cellular growth in TNBC and HER2+ BC. While the effects of nebivolol on cell growth inhibition are not mediated by β3 receptors, it is possible that other effects of nebivolol on invasion/migration and mitochondrial oxidative phosphorylation are dependent on these pathways. In-depth future analysis including detailed *in vivo* studies are required to further validate these results. Also, *in vivo* pharmacokinetic studies investigating the tumoral levels of nebivolol are needed to determine whether the doses that are used in patients with hypertension are sufficient to achieve the desired anti-tumor effects. Our studies also highlight the need to investigate individual β-blockers separately due to differences in their affinity, potency, and efficacy against different β-adrenergic receptors. Large real-world dataset is also needed to investigate the effects of nebivolol on cancer-specific and all-cause mortality, especially in HER2+ and TNBC subgroups. Future analysis of nebivolol in early-stage disease setting with larger sample size (currently not available) and in non-hypertensive patients will be of interest. Further, our studies highlight that a similar approach can be used to identify potential drug candidates for repurposing in other cancer types.

## Data Availability

The original contributions presented in the study are included in the article/Supplementary Material, further inquiries can be directed to the corresponding author.
