# Supplementary material for: Screening of GPCR drugs for repurposing in breast cancer
Source: Front Pharmacol. 2022 Dec 6;13:1049640. doi: 10.3389/fphar.2022.1049640 (PMC9763283; doi:10.3389/fphar.2022.1049640)

Supplementary file 6. Nebivolol did not inhibit cell growth of the normal human mammary epithelial cell line MCF10A.

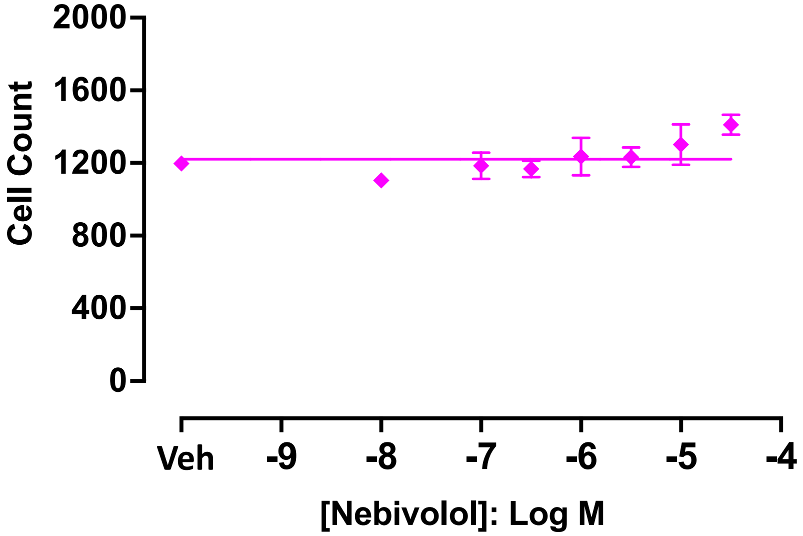

Supplement: Supplementary file 1 [file DataSheet2.PDF]
