# Supplementary material for: Screening of GPCR drugs for repurposing in breast cancer
Source: Front Pharmacol. 2022 Dec 6;13:1049640. doi: 10.3389/fphar.2022.1049640 (PMC9763283; doi:10.3389/fphar.2022.1049640)
Supplement: Supplementary file 4 [file DataSheet3.PDF]

Supplementary file 7. The inhibition of mitochondrial respiration by Nebivolol. Seahorse analysis of SUM159 cells suggesting a decreased oxygen consumption rate (OCR) after treatment with 10 uM Nebivolol. A Seahorse XFp analyzer was used for the analysis. The data was obtained from three replicates and the graph represents mean +/- standard error of mean.

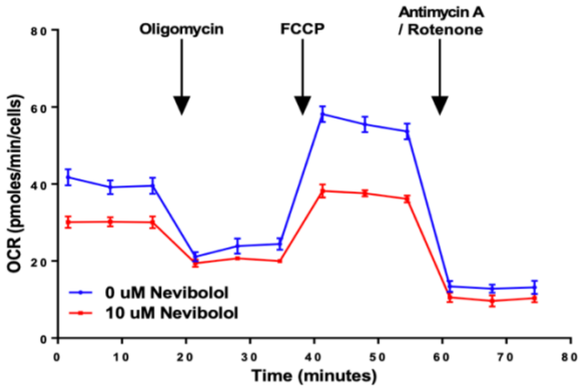

Assay Well Parameter Calculations

Group Name	Assay Wells	Non-Mitochondrial Oxygen Consumption	Basal Respiration	Maximal Respiration	Proton Leak	ATP Production	Spare Respiratory Capacity	Spare Respiratory Capacity as a %	Coupling Efficiency	Acute Response
Control	B01	13.80	26.05	42.29	7.74	18.31	16.24	162.35%	70.27%	
	C01	13.00	28.46	47.21	9.06	19.40	18.75	165.88%	68.17%	
	D01	11.65	25.66	46.40	8.11	17.56	20.74	180.80%	68.41%	

Group Name	Assay Wells	Non-Mitochondrial Oxygen Consumption	Basal Respiration	Maximal Respiration	Proton Leak	ATP Production	Spare Respiratory Capacity	Spare Respiratory Capacity as a %	Coupling Efficiency	Acute Response
10 uM Nebivolol	E01	10.53	18.97	29.60	9.48	9.49	10.63	156.05%	50.03%	
	F01	7.97	23.82	29.50	10.39	13.43	5.68	123.85%	56.37%	
	G01	8.09	14.29	20.56	7.36	6.93	6.26	143.80%	48.50%	
