# Supplementary material for: Screening of GPCR drugs for repurposing in breast cancer
Source: Front Pharmacol. 2022 Dec 6;13:1049640. doi: 10.3389/fphar.2022.1049640 (PMC9763283; doi:10.3389/fphar.2022.1049640)
Supplement: Supplementary file 5 [file DataSheet1.PDF]

Supplementary file 5. FDA-approved drug candidates and their GPCR targets, indications and 2 most common side effects (incidence)

Drug	GPCR targets	Indication	2 most common side effects (incidence)
Cinacalcet HCl	(CaS, mGlu5) ^{Ag}	Hyperthyroidism<br>Parathyroid Carcinoma	Nausea (66%),<br>Vomiting (52%)
Nebivolol	(β1, β2, β3) ^{Antg}	Hypertension	Headache (7%),<br>Fatigue (5%)
Pimavanserin	5-HT2A^{InAg}	Hallucinations<br>Delusions	Peripheral edema (7%),<br>Confusion (6%)
Vortioxetine (Lu AA21004) HBr	(5-HT1A, 5-HT1B) ^{PaAg}, (5-HT5A , 5-HT7, 5-HT2A, 5-HT6) ^{Antg}	Major depressive disorder	Nausea (32%),<br>Diarrhea (7%)

Ag: Agonist; Antg: Antagonist; InAg: Inverse Agonist; PaAg: Partial Agonist,
